# Estimating SARS-CoV-2 variant fitness and the impact of interventions in England using statistical and geo-spatial agent-based models

**DOI:** 10.1098/rsta.2021.0304

**Published:** 2022-10-03

**Authors:** Robert Hinch, Jasmina Panovska-Griffiths, William J. M. Probert, Luca Ferretti, Chris Wymant, Francesco Di Lauro, Nikolas Baya, Mahan Ghafari, Lucie Abeler-Dörner, The COVID-19 Genomics UK (COG-UK) Consortium, Christophe Fraser

**Affiliations:** ^1^ Big Data Institute, Li Ka Shing Centre for Health Information and Discovery, Nuffield Department of Medicine, University of Oxford, Oxford, UK; ^2^ The Queen's College, University of Oxford, Oxford, UK; ^3^ Department of Zoology, University of Oxford, Oxford, UK

**Keywords:** SARS-CoV-2, agent-based model, statistical model, virus variants, vaccinations, geo-spatial model

## Abstract

The SARS-CoV-2 epidemic has been extended by the evolution of more transmissible viral variants. In autumn 2020, the B.1.177 lineage became the dominant variant in England, before being replaced by the B.1.1.7 (Alpha) lineage in late 2020, with the sweep occurring at different times in each region. This period coincided with a large number of non-pharmaceutical interventions (e.g. lockdowns) to control the epidemic, making it difficult to estimate the relative transmissibility of variants. In this paper, we model the spatial spread of these variants in England using a meta-population agent-based model which correctly characterizes the regional variation in cases and distribution of variants. As a test of robustness, we additionally estimated the relative transmissibility of multiple variants using a statistical model based on the renewal equation, which simultaneously estimates the effective reproduction number *R*. Relative to earlier variants, the transmissibility of B.1.177 is estimated to have increased by 1.14 (1.12–1.16) and that of Alpha by 1.71 (1.65–1.77). The vaccination programme starting in December 2020 is also modelled. Counterfactual simulations demonstrate that the vaccination programme was essential for reopening in March 2021, and that if the January lockdown had started one month earlier, up to 30 k (24 k–38 k) deaths could have been prevented.

This article is part of the theme issue ‘Technical challenges of modelling real-life epidemics and examples of overcoming these’.

## 1. Introduction

The first year of the COVID-19 pandemic was characterized by multiple epidemic waves, linked to the evolution and spread of different SARS-CoV-2 variants across multiple countries. While the initial wave in Europe and the USA was driven by a large number of introductions from multiple lineages [1,2], the spread of the lineages behind the second wave was facilitated by travel and tourism, especially in the summer of 2020. Founder effects and incomplete genetic surveillance make it difficult to disentangle the epidemiological factors contributing to the rapid spread of specific variants, such as B.1.177, which was the most common variant of the European epidemic during the autumn of 2020 [3]. However, variants with a strong fitness advantage dominate, as with Alpha, which has been shown to have a clear reproductive advantage through multiple approaches [4–6]. At the time of writing, there is not yet sufficient evidence as to what the reproductive advantage of B.1.177 was with respect to previous SARS-CoV-2 lineages.

The UK is a natural case study for understanding the spatial and epidemiological dynamics of these variants. It has had one of the most comprehensive national genomic surveillance programmes—the COVID-19 Genomic UK Consortium (COG-UK)—which contributed about half of the SARS-CoV-2 genomic sequences publicly available worldwide in 2020 [7]. Furthermore, the Alpha lineage was first detected in the southeast of the UK and its spread occurred mostly through local transmissions rather than introductions [6], providing constraining observations for models of geographical spread of variants.

One of the most relevant metrics that inform epidemic response is the effective reproduction number, *R*. Monitoring whether *R* is greater than or less than 1 is key to understanding whether the epidemic is under control, and monitoring changes in *R* in response to interventions allows us to assess their impact [8,9]. Moreover, risk assessment of different variants of interest considers their relative transmissibility, measured for example as the ratio of their effective reproduction numbers [4]. The renewal equation relates the exponential growth rate of the epidemic to *R* via the generation-time distribution of the pathogen (i.e. the time between being infected and infecting others) [10,11], providing an effective framework to infer reproduction numbers [11] and to model the impact of non-pharmaceutical interventions such as contact tracing [12–14]. Several Bayesian inference methods are available that are well suited to general-purpose near-real-time estimation [15], including the widely used *EpiEstim* [16] and more recent approaches developed during the COVID-19 pandemic, such as *epidemia* [17] and *EpiNow2* [18], which treat the epidemic dynamics as latent states.

Mathematical modelling has been used extensively throughout the COVID-19 pandemic to quantify understanding [13,19] and inform response [20–22]. There are multiple types of mathematical models for epidemics [23]. While renewal-equation models can infer *R*(*t*) and provide nowcasts, they suffer from several limitations when modelling heterogeneous populations, geographically structured epidemics and complex interventions (especially if they span multiple transmissions, such as contact tracing). These limitations can be overcome using agent-based models (ABMs), also known as individual-based models, which model individuals and their behaviours. ABMs provide a high degree of flexibility in modelling dynamics and control interventions [24–27]. When considering epidemics in large regions, it may be necessary to model human movements (e.g. commuting and international travel) and seeding events at multiple loci [28]. Approaches to incorporating spatial spread include statistical models [29], meta-population models [30] and ABMs [31].

The main aim of this work was to understand the spatial–temporal characteristics of the SARS-CoV-2 epidemic in England, during a period in which new viral variants were seeded and many public interventions to prevent the spread of the virus were implemented. To model all these effects explicitly requires a complex geo-spatial ABM; however, such models contain large numbers of parameters, so the challenge is to apply these models in a statistically robust way. One question of particular interest was whether the spatial–temporal characteristics of the epidemic in England could be explained by only the relative fitness of the Alpha variant and its seeding at a single location, without requiring specific regional–temporal factors (noting that during the Alpha wave all restrictions in England were national not regional). As a test of the robustness of the ABM and the parameter estimation method, we estimated the relative transmissibility of different viral variants using both the ABM and a simple renewal-equation model, which had a small number of parameters for which posterior distributions could be calculated. Finally, with a well-calibrated geo-spatial ABM, we aimed to estimate the impacts of lockdown and vaccination, including any geographical heterogeneity, during a period when multiple variants were circulating at different levels in each region.

We modelled the genetic and geographic heterogeneity of the second epidemic wave in England using both renewal-equation and agent-based modelling approaches. First, we developed a Bayesian model based on the renewal equation, which provides a statistical estimate of both *R*(*t*) and the relative transmissibility of the circulating variants. We then extended an existing ABM, OpenABM-Covid19 [26], to include multiple virus variants and vaccinations. A meta-population model was constructed using the ABM to account for regional variation in epidemic dynamics. Importantly, the model does not contain time-dependent regional parameters, so regional variations in the epidemic curves are driven by the geographical spread of variants. Posterior distributions for key parameters, such as variant transmissibility, were estimated using approximate Bayesian computation (ABC), with the estimates of the renewal-equation model informing the priors. Finally, we used the calibrated ABM model to simulate the spatial spread of the B.1.177 and Alpha variants that dominated England in the autumn and winter of 2020 and early 2021 under three different scenarios, to analyse the vaccine programme and timing of lockdowns.

## 2. Methods

### (a) Multi-variant renewal-equation model

We present a statistical model for estimating the relative transmissibility of variants using the renewal equation. The key assumption in the model is that the relative transmissibility of different variants is constant over time even as the absolute transmissibility in a population varies due to interventions and prior infections. We also assumed that the number of new infections with each variant is independent of the infections with the other variants. Denoting the underlying number of new infections of variant *i* at discrete time *t* by $I_{i}(t)$ and the reproduction number of the base variant by R(t), the renewal equation for each variant is

$$I_{i}(t)=S_{i}(t)+R(t)\beta_{i}\sum_{g=1}^{g_{m}}G(g)I_{i}(t-g),$$

where $\beta_{i}$ is the relative transmissibility of the variant, G(g) is the generation-time distribution, which we modelled as a discretized gamma distribution, $g_{m}$ is the maximum time for which someone is infectious and the $S_{i}(t)$ are seeding events. Seeding events refer to the external introduction of infections and are modelled to occur at specified times, with the rate of seeding being an estimated parameter. The underlying reproduction number is expected to change slowly with time but to exhibit sudden changes at the start and end of lockdowns; therefore we modelled the reproduction rate as a lognormal process with discontinuities at the start and end of lockdowns. New infections are only counted as cases once the individual has had a positive PCR or antigen test, which normally occurs following the appearance of symptoms. Denoting the number of new cases of variant *i* at time *t* by $C_{i}(t)$, the expected number of new cases is

$$E(C_{i}(t))=A_{c}\sum_{s=1}^{s_{m}}T(s)I_{i}(t-s),$$

where T(s) is the distribution of the time from infection to obtaining a positive test, which we modelled as a gamma distribution using parameters estimated for the incubation-period distribution with the mean increased by 1 day (the assumed delay between onset of symptoms and testing), $A_{c}$ is the case ascertainment rate, which we assumed to be constant over the time period and the same for all variants, and *s_m_* is the assumed maximum time before being tested. Finally, we used an observation model to link the observed number of cases to the expected number estimated by the infection model

$$C_{i}(t)=\text{NegBinomial}(E(C_{i}(t)),\phi_{\mathrm{OD}}),$$

where $\phi_{\mathrm{OD}}$ is the over-dispersion parameter. Range priors were put on all the parameters and the posterior distribution was sampled using the default Markov chain Monte Carlo sampler in Stan [32]. Full details of the priors and technical specifications are given in the electronic supplementary material, and the code is available as an R package on GitHub (https://github.com/BDI-pathogens/VariantREstimate).

### (b) Multi-variant agent-based modelling

OpenABM-Covid19 is an ABM for the transmission of SARS-CoV-2 and was developed to model contact tracing and other non-pharmaceutical interventions in early 2020 [20,21,26]. For the present work, we extended this model to simulate the subsequent infection waves by introducing novel viral variants and vaccinations.

New viral variants enter simulations via seeding events, where susceptible people are infected with the new variant (from an external source). A newly infected person inherits the same variant as their infector. Simultaneous co-infection with different variants is not modelled; nor is the evolution of existing variants into new ones. Each new variant has a transmissibility multiplier relative to the base infectious rate and a disease severity multiplier that changes the fraction of people who are hospitalized (and thus the fatality rate). Upon recovery from an infection, immunity to other variants is conferred upon the individual according to a cross-immunity matrix. If cross-immunity is conferred to another variant, it is polarizing (i.e. the same for multiple challenges) and wanes at the same rate as that of the infecting variant. With more than three variants, cross-immunity between variants is assumed to be fully correlated, i.e. if somebody gains immunity to a variant where the cross-immunity is 60%, then they will also have immunity to all variants where the cross-immunity is greater than 60% (see electronic supplementary material for a worked example). All immunity from infection is assumed to be sterilizing immunity which prevents re-infection (i.e. not just reduction in symptoms or severity).

### (c) Modelling vaccination against COVID-19

An important development in the control of SARS-CoV-2 has been the introduction of vaccinations from late 2020 onwards. While vaccines have been incredibly successful at preventing hospitalizations and deaths, their effectiveness against mild symptoms and preventing transmission has been lower [33–35]. To model this, we introduce different levels of immunity: immunity against severe symptoms, immunity against symptoms and sterilizing immunity. While all three types of immunity prevent hospitalization and death, only sterilizing immunity completely prevents infection and thus onward transmission. Immunity against symptoms means that the individual will be infected but asymptomatic, so they will transmit the virus at a lower rate than somebody who develops symptoms (with the model's normal reduced transmissibility of asymptomatic infections relative to symptomatic ones).

The model supports multiple vaccines, where each vaccine has the ability to confer each type of immunity to each variant at different levels of effectiveness. Immediately after a vaccine is administered, there is a gap (modelled as 14 days) before immunity is conferred, and immunity is kept for a set time. The modelled effect of vaccines only ever increases or leaves unchanged any pre-existing immunity (i.e. vaccination after natural infection would not decrease the immunity that infection conferred).

### (d) Geo-spatial meta-population modelling

Certain aspects of viral transmission can only be understood by modelling geographic spread. For example, the Alpha variant first appeared in Kent in the southeast of England in September 2020 but did not spread to the north of the country until December 2020. In the time between the initial introduction of Alpha and geographic spread to the north of England, there was a national lockdown in England, which further complicated the dynamics of the spread of the variant. To understand the factors affecting these dynamics, we developed a meta-population model of OpenABM using census data from the Office for National Statistics to calibrate movements.

Meta-population models combine local infection transmission models in isolated populations with movement models, allowing for cross-population transmissions [28]. Compared to large single-population spatially structured models, meta-population models clearly split the concepts of the infection dynamics and geographic movement, simplify model calibration, and allow numerical simulations to be efficiently parallelized. In our model, we first split England into 149 domains by the upper-tier local authority (UTLA). Each UTLA has between about 100 000 and 1 000 000 people, and both census and SARS-CoV-2 case data are published at this level. In each domain, we ran a separate OpenABM model. When modelling SARS-CoV-2, we are primarily interested in the effects of commuters, shoppers, family visits and day trips on spreading the infection, as opposed to long-term relocations. Therefore, we used a Lagrangian model where individuals travel from their home domain to others for a time before returning home [23,36]. We modelled each inter-domain movement as occurring only during a single day, and these interactions are in addition to the interactions of the individual in their home domain. Under the additional assumption that all cross-population interactions occur between random people in each domain, it can be shown (see electronic supplementary material) that the expected number of transmissions in domain *n* from domain *m* is

$$E(\text{infected}\;\text{in}\;n\;\text{from}\;m\;\text{on}\;t)\approx {\bar{S}}_{n}(t)\alpha \phi_{m,n}\sum_{k=1}^{k_{m}}G(k)I_{m}(t-k),$$

where ${\bar{S}}_{n}$ is the mean susceptibility of individuals in domain *n*, α is a measure of the strength of cross-border interactions, $\phi_{m,n}$ is the total number of cross-border interactions between the domains normalized by the population in domain *m*, G(k) is the generation-time distribution and $I_{m}(t)$ is the number of infections in domain *m* at time *t*. This is implemented in the meta-population model by calculating the expected number of people who would be infected in each domain if the entire population were susceptible, and then challenging that number of individuals randomly selected from the population (those with immunity will not be infected). In response to non-pharmaceutical interventions such as lockdowns and social distancing, the value of α varies with time and was calibrated using the Google mobility data for transit [37]. Finally, each variant is treated independently, with cases for each variant being seeded between regions based on the number of new infections of the variant.

Cross-border flows were estimated using 2011 UK census data on individuals who reside and work in different local authority areas (dataset WF01BEW [38]) for the daily commuting component, and on internal migration matrices (table IM2020-T7B [39]) for estimating the relative flows of family and friend visits between regions. For the central London boroughs (e.g. the City), the majority of external interactions occur between people who are both from external regions, so a proportion of interactions were pooled and modelled as interaction directly between the external regions (see electronic supplementary material). The relative contribution of the flow matrices and the fraction of cases caused by migrations were estimated as part of the overall model calibration.

### (e) Simulation of the second wave of COVID-19 in England

We modelled the trajectory of the wild-type (defined as all early lineages which were assumed to have the same transmission properties), B.1.177 (and all sublineages) and Alpha lineages in England between September 2020 and May 2021. For computational speed and memory requirements, OpenABM-Covid19 was run using static contact networks (requiring up to 20 times less CPU time; see [26] for detailed analysis), and 11 million agents were modelled (20% of the English population), with the results scaled up when comparing with data. To model the natural immunity from the March 2020 epidemic, a geographically uniform epidemic was allowed to spread in the population to achieve a prevalence of 7% (population survey estimate was 5.21–8.64% [40]) before being eliminated by a strong lockdown. Infections of the wild-type and B.1.177 lineages were then randomly seeded in the population at a constant rate for a month prior to the start of the simulation period in September 2020. The relative number of seeds in each lineage was chosen to match COG-UK data [41], and the number in each region was chosen to match the regional case variation at the start of September 2020. The number of contacts relative to pre-epidemic levels was estimated using Google mobility data. The Alpha variant was seeded between 20 September 2020 and 4 October 2020 in the Kent and Medway UTLAs based on the genomic surveillance data [41]. Seeding was modelled by randomly infecting agents in these areas on each day of the seeding period, with the seeding rate estimated in the calibration (see §2f). Given that static contact networks were used, reduction in the number of contacts was modelled by reducing the probability of transmission in any interaction between two individuals on the relevant network (equivalent in expectation to randomly dropping that number of interactions assuming independence). A meta-analysis of six studies of the efficacy of mask wearing estimated that it reduces incidence by 25–71% [42]. During the period of study, mask wearing in England on public transport and in shops was mandated, and over 95% of individuals surveyed reported wearing a mask at some time [43], although not in classrooms or workplaces. Therefore, we used the lower end of the meta-analysis range and modelled the overall effect as a 25% reduction in transmission rates on all non-household networks. UK survey estimates in January 2021 for duration-weighted adherence to self-isolation were 40.8–62.8% [44]; therefore 60% of people were assumed to isolate at home upon developing symptoms, with a 2% daily drop-out. The reduction in mobility from the Google mobility data was not sufficient to explain the reduction in infections during the nationwide lockdowns in November 2020 and January 2021, possibly because of other changes in behaviour such as reduced socializing. Therefore, an extra factor to reduce non-household contacts during these lockdowns was applied equally to all regions and was estimated in the calibration (see §2f). The growth of the epidemic varied in each region, so a single relative transmission multiplier for each region was applied to the entire period of the epidemic. Local lockdown measures in October 2020 were not modelled. The vaccine programme in England started in December 2020 and was highly targeted by age (see electronic supplementary material).

### (f) Model calibration

The baseline OpenABM-Covid19 parameters were used in each of the English UTLA regions with the population scaled by 20% [26]. The additional 18 new parameters for the meta-population model were estimated using ABC [45,46]. Flat range priors were set on each parameter (see electronic supplementary material) and 2000 parameter sets were sampled from the prior using Latin hypercube sampling [47,48]. Five stochastic replicate runs of the model were generated for each parameter set and summary statistics were calculated for seven features (see electronic supplementary material). The top 25 parameter sets were then used to seed 25 chains and sequential ABC was performed (see electronic supplementary material). The posterior distribution for the time series of cases and deaths was taken from all the stochastic replicates of these parameter sets.

### (g) Modelling different epidemic scenarios

Hypothetical scenarios were simulated using the calibrated model by changing the interventions. Two policies were assessed: the vaccine programme and relaxation of restrictions in December 2020 following the second national lockdown in November 2020. To achieve this, we simulated three scenarios: (i) no vaccinations, but the second and third national lockdowns as they occurred; (ii) starting the third national lockdown immediately after the second, but having it last only one month; and (iii) starting the third lockdown immediately after the second lockdown finished, keeping it at the same length as the actual third lockdown (see charts in figure 3 for schematic of scenarios). The actual third lockdown was stricter than the second (e.g. schools were closed), so all lockdowns in the scenarios after the end of the actual second lockdown were modelled using these stricter rules. For each of these scenarios, we ran simulations using all the parameter sets accepted by the sequential ABC calculation to obtain posterior distributions of cases and deaths.

## 3. Results

### (a) Relative transmissibility of viral variants

The transmissibility of the B.1.177 and Alpha lineages relative to the wild-type lineages was first estimated by fitting the renewal-equation model to weekly cases by lineage. The transmissibility of the B.1.177 variant relative to the wild-type lineages was estimated as 1.14 (credible interval 1.12–1.16), suggesting that only a modest increase in fitness was sufficient for it to grow dramatically relative to the wild-type lineages over this period (electronic supplementary material, figure 1*a*). The fraction of the B.1.177 lineage relative to wild-type lineages in England continued to increase for four months after the initial seeding events (until the sweep of the Alpha variant), suggesting that the increase cannot be understood purely in terms of seeding one specific age group. The transmissibility of the Alpha variant relative to the wild-type lineages was estimated as 1.71 (1.65–1.77; electronic supplementary material, figure 1*b*) and as 1.51 (1.44–1.54; electronic supplementary material, figure 1*c*) relative to B.1.177, which is consistent with previous estimates using other methods (1.43–1.90 [5] and 1.5–2.0 [4], both defined relative to the mixed population of other contemporaneous lineages). The posterior distribution of the wild-type reproduction number and overall reproduction number are shown in the electronic supplementary material, figure 1*d*. Note that the wild-type reproduction number falls monotonically, with the exception of the jump at the end of the November lockdown, and it is less than 1 throughout December 2020. The renewal-equation model assumes a static case ascertainment rate; however, at times of high incidence the ascertainment rate may fall due to a lack of test availability. A sensitivity analysis demonstrated that the estimates of relative transmissibility are insensitive to dynamic changes in case ascertainment (see electronic supplementary material). Furthermore, a comparison of case data [49] with prevalence survey data [50] shows no evidence of a change in the case ascertainment rate over the period of this study (see electronic supplementary material).

**Figure 1.**
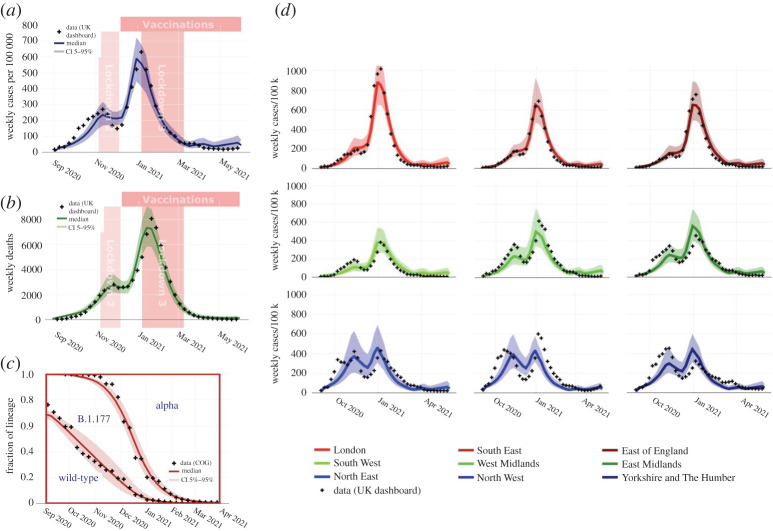
Nationwide and regional epidemic time series. (*a*,*b*) The time series of the ABM ABC posterior of cases and deaths, respectively, along with the actual data [49]. (*c*) The time series of the model posterior of the fraction of cases by lineage along with the raw COG-UK data [41]. (*d*) The weekly cases per 100 k population in each of the nine English regions (electronic supplementary material, figure 3) along with the actual data [49]. The model correctly captures the shape of the curves in each region despite not containing region-specific dynamic parameters. (Online version in colour.)

The relative transmissibilities of B.1.177 and Alpha were also inferred by ABC for the geo-spatial ABM. The posterior distributions are approximately bell-shaped with means of 1.16 and 1.73, respectively, shown alongside the estimates from the renewal-equation model in the electronic supplementary material, figure 1*a,b*. The transmissibility of Alpha versus B.1.177 was estimated at 1.51, with the posterior distributions of the two models being almost identical (electronic supplementary material, figure 1*c*). The two models have consistent estimates for the relative transmission strengths despite being vastly different methods, and despite using only weakly informative priors (i.e. both datasets are informative and concordant). The model estimate of the nationwide fraction of each lineage was calculated and reproduced the growth of the B.1.177 lineage relative to the wild-type lineages in autumn 2020 and the sweep of the Alpha lineage in late 2020 (figure 1*c*, compared with COG-UK data). To demonstrate the goodness of fit to the basic properties of the epidemic, the number of weekly cases per 100 000 population and deaths are plotted in figure 1*a*,*b* along with the publicly reported values [49].

### (b) Geographic variation of cases and variants

Both the model estimates and the actual data at the level of a single UTLA are noisy, so we only compare data at an aggregated regional level (there are nine English regions each with 5–10 million inhabitants; see electronic supplementary material, figure 3). Figure 1*d* shows good agreement between model estimates and the publicly reported data for the number of cases by region. The dynamics of cases are different in each of the regions. In the three regions closest to Kent (London, South East and East of England, shown in the top panels in red), cases grew steadily in September and October before plateauing during the November lockdown. Cases then rose very steeply in December 2020 after the lockdown was lifted, peaking at a much higher value around the end of December and the start of January. In the three northern English regions (North East, North West and Yorkshire, shown in the bottom panels in blue), cases rose much more rapidly in September and October of 2020, then declined during the November 2020 lockdown, before rising to a second similarly sized peak around the end of December and the start of January. This difference in dynamics is due to the geographic spread of the Alpha variant: there are no region-specific dynamic parameters in the model. The November lockdown was not as strict as the January lockdown (e.g. no school closures), and while it caused infections in the B.1.177 lineage to fall, it could not prevent infections of the Alpha lineage increasing. In November, almost all the cases of the Alpha variant occurred in the southeast of England, which explains why cases did not reduce in these regions during the November lockdown.

**Figure 2.**
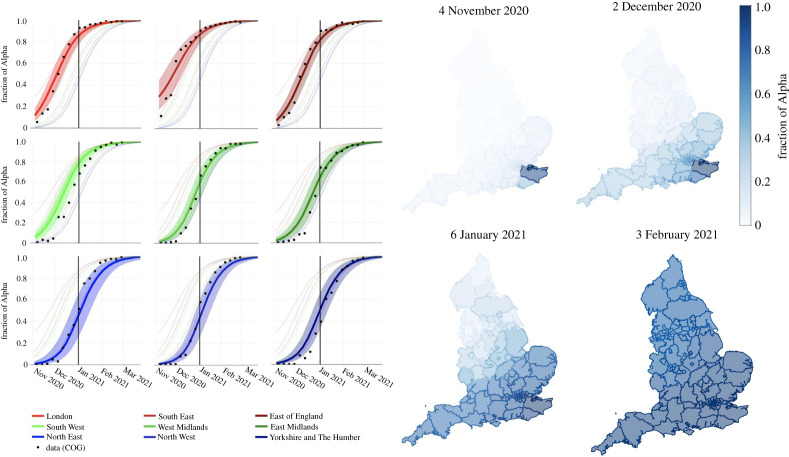
Geographic spread of the Alpha variant. The ABM ABC posterior of the fraction of new cases that were the Alpha variant, shown over time and English region. The left set of panels show the nine regions of England, with COG-UK data as black dots and the model posterior in colour (the median is represented by a thick line and the central 90% of the posterior with shading; the median for every region is replotted in every panel with faint lines to highlight the timing difference between regions). The timing of the sweep in each region is accurately predicted by the ABM (despite it not containing region-specific dynamic parameters), with the exception of the South West, where it occurred about two weeks too early in the model. The right set of panels show the model results at finer geographical resolution (ULTAs) at the four time points indicated. (Online version in colour.)

The model estimates a rapid spread of the Alpha variant across the country during December 2020 and early January 2021 (figure 2), in agreement with analysis of the COG-UK data [5]. The fraction of cases of the Alpha lineage is plotted for each region and is compared with the COG-UK data in figure 2. The relative timing and speed of the sweep to the Alpha variant are captured by the model for all regions except for the South West, where it occurred about 10 days earlier in the model than in the actual data. The January 2021 lockdown was implemented before the sweep to Alpha had occurred in the northern English regions, causing them to experience a dramatically smaller second wave than the southern regions.

**Figure 3.**
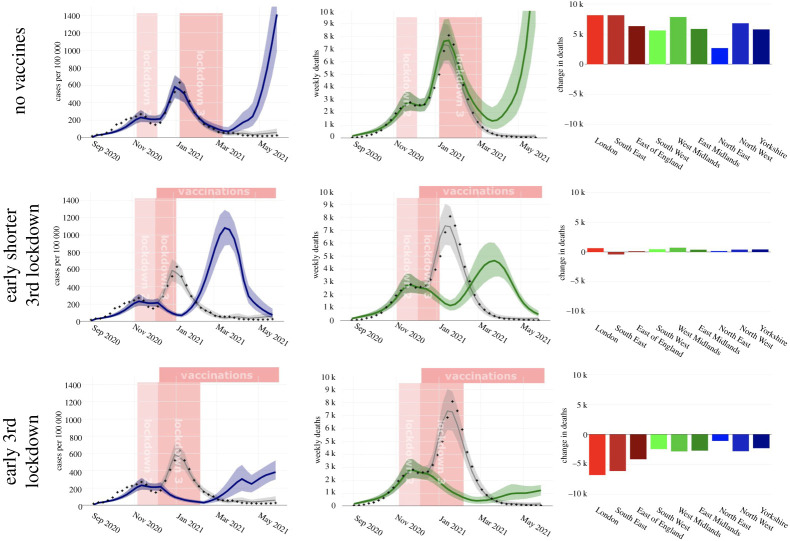
Scenario analysis: each row contrasts the central modelled scenario (in which interventions were as in reality) with a different counterfactual scenario. The left column shows weekly cases per capita over time, with the actual scenario in grey and the counterfactual in blue. The central column shows total deaths over time, with the actual scenario in grey and the counterfactual in green. The right column shows the difference in total deaths by region from December 2020 to May 2021 for the counterfactual scenario compared with the actual scenario. In the top row, the counterfactual scenario considered is no vaccine programme. In this counterfactual, deaths would have been higher and a new wave would have occurred following the easing of lockdown in March 2021. The second row shows the counterfactual scenario in which the third lockdown was brought forward to December 2020 but only lasted a month. The results demonstrate that this would have just delayed the timing of the second wave by a month. The third row shows the counterfactual scenario in which the third lockdown was brought forward to December 2020 and lasted the same length of time. The results demonstrate that 30 k deaths might have been prevented because the spread of Alpha would have been postponed until after vaccination of the most vulnerable, and that these prevented deaths would have been concentrated in the South East and London where the Alpha variant dominated first. (Online version in colour.)

### (c) Importance of the vaccine programme

The roll-out of the vaccine programme in England starting in December 2020 has been heralded as pivotal in preventing both deaths and infections. Our simulations of the effect of the vaccination programme—comparing outcomes in the presence and absence of vaccination—confirm this (figure 3, top row). Specifically, we show that while cases and deaths did fall during the January lockdown, without the vaccination programme deaths would have still been at 2000 per week at the end of March 2021, compared with the actual figure of 200 per week. Furthermore, without the vaccine programme, both cases and deaths would have started to rise again from mid-March 2021 as the lockdown started to be eased. Hence, vaccination played an important part in curbing the second wave and resurgence from Alpha after the reopening in March 2021.

### (d) A December lockdown could have prevented deaths

Results comparing the impact of starting the third national lockdown in December 2020 instead of January 2021, and of it lasting either one month or two months, under two simulation scenarios are shown in figure 3 (middle and bottom row). In the scenario where the December lockdown lasted only a month, we observe a large increase in cases in March 2021 with a simultaneous large increase in deaths (figure 3, second row); this scenario would have only delayed deaths. However, in the scenario where the December lockdown lasted for two months, while cases rose after the lockdown ended at the start of February, the number of deaths rose only gradually and remained below 1000 a week. This is because the majority of the most vulnerable people would have been vaccinated by the time cases started to rise, with the vaccination breaking the chain between cases and deaths. We estimate that in this scenario, compared with the lockdown strategy that was actually used, total deaths would have been approximately 30 k (24 k–38 k) lower between January and May 2021, with approximately 17 k of these averted deaths occurring in London, the South East and the East of England.

## 4. Discussion

We analysed the relative fitness of three SARS-CoV-2 variants using a renewal-equation statistical model and a geographically detailed ABM. The two models were in close agreement with respect to their estimates of the transmissibility of the B.1.177 and Alpha variants relative to the wild-type lineages. The ABM was able to reconstruct the shapes of the epidemic curves seen in the different English regions despite not containing any time-dependent regional parameters, suggesting that the differences could be explained by the geographical spread of the Alpha variant from the southeast corner of England. We estimated the transmissibility of Alpha relative to B.1.177 to be 1.51 (credible interval 1.44–1.54), consistent with previous analyses of the relative transmissibility of Alpha [4,5]. Interestingly, our results suggest some fitness advantage for B.1.177 relative to previously circulating lineages, with an increase of 14% (12–16%) in transmissibility. An earlier analysis [3] concluded that B.1.177 has no significant transmission advantage, although it did not exclude the possibility of a small advantage. The model of [3] was discrepant with the final fraction of the B.1.177 lineage by up to 12-fold. Another genomic analysis of the B.1.177 lineage in England showed it had a small advantage relative to the other lineages in September 2020 [51], although the advantage was not seen in all of the sublineages of B.1.177. However, almost 80% of all the B.1.177 samples sequenced by COG-UK [41] were of the main lineage, so this result is consistent with our findings.

We also presented ABM simulations of counterfactual scenarios based on the model calibration performed previously. Our simulations of a counterfactual scenario without vaccines highlight the key role of the vaccination campaign in enabling gradual reopening in March without a significant increase in cases and deaths. These results provide further evidence that the vaccination programme saved thousands of lives in January to May 2021 and was an essential requirement for the lockdown to be eased in March 2021, despite doubts raised in the past [52]. Our counterfactuals also confirm the rule of thumb that earlier interventions are better than stronger or longer ones [53,54]. For example, a shorter but earlier lockdown in December 2020–January 2021 would have been as effective as the actual UK lockdown of January–March 2021, and an earlier lockdown of the same length could have prevented up to 30 k deaths between January and May 2021, concentrated in London and the South East of England where the Alpha variant first became dominant.

The ABM presented here could be extended in different directions. One of the most relevant extensions for future investigations would be an even more realistic model of immunity, in particular regarding the waning of vaccine protection. The rate of waning is expected to differ by age and by the type of immunity in question (against infection, symptoms or more severe outcomes). For example, while the efficacy of the Pfizer–BioNTech vaccine against symptoms from the Delta variant has been shown to drop from about 90% to 70% after 20 weeks, protection against hospitalization remains above 90% [34], suggesting that part of the waning is a decrease in the level of protection. Additionally, in populations with high levels of vaccinated people but where the virus is still circulating, modelling the effect on immunity of asymptomatic or mild infections in vaccinated people might be necessary. If new variants arise in the future, the model could also be extended to include more complex cross-immunity between multiple variants or between variants and vaccines. These issues are critical in determining the epidemic's dynamics in a multi-variant world, and their inherent complexity requires agent-based modelling to address. The robust application of ABMs to SARS-CoV-2 and other pathogens will therefore become an increasingly important tool for global health.

## Data Availability

Full details of the priors and technical specifications are given in the electronic supplementary material, and the code is available as an R package on GitHub (https://github.com/BDI-pathogens/VariantREstimate). Electronic supplementary material is available online [56].
